# Towards a Hybrid Energy Efficient Multi-Tree-Based Optimized Routing Protocol for Wireless Networks

**DOI:** 10.3390/s121217295

**Published:** 2012-12-13

**Authors:** Nathalie Mitton, Tahiry Razafindralambo, David Simplot-Ryl, Ivan Stojmenovic

**Affiliations:** 1 INRIA Lille-Nord Europe, 59650 Villeneuve d’Ascq, France; E-Mails: tahiry.razafindralambo@inria.fr (T.R.); david.simplot-ryl@inria.fr (D.S.-R.); 2 SITE, University of Ottawa, Ottawa, ON K1N 6N5, Canada; E-Mail: ivan@site.uottawa.ca

**Keywords:** geographic routing, guaranteed delivery, energy efficiency

## Abstract

This paper considers the problem of designing power efficient routing with guaranteed delivery for sensor networks with unknown geographic locations. We propose HECTOR, a hybrid energy efficient tree-based optimized routing protocol, based on two sets of virtual coordinates. One set is based on rooted tree coordinates, and the other is based on hop distances toward several landmarks. In HECTOR, the node currently holding the packet forwards it to its neighbor that optimizes ratio of power cost over distance progress with landmark coordinates, among nodes that reduce landmark coordinates and do not increase distance in tree coordinates. If such a node does not exist, then forwarding is made to the neighbor that reduces tree-based distance only and optimizes power cost over tree distance progress ratio. We theoretically prove the packet delivery and propose an extension based on the use of multiple trees. Our simulations show the superiority of our algorithm over existing alternatives while guaranteeing delivery, and only up to 30% additional power compared to centralized shortest weighted path algorithm.

## 1. Introduction

Wireless *ad hoc* networks, especially sensor networks, have received a lot of attentions in recent years due to their potential applications in various areas such as monitoring, security and data gathering. However, they have some limitations compared with wired infrastructure networks. Energy consumption and scalability are two challenging issues when designing sensor network protocols such as routing protocols since they operate on limited capacity batteries while the number of deployed sensors could be very large.

Position awareness in sensor networks improves the efficiency of route discovery and broadcasting algorithms. The fundamental idea behind position awareness (referred also as *geographic* or *geometric* information) is to provide a global position information to each node in the network. This information can be obtained through devices such as GPS or Galileo. Protocols using geographic information for routing (Cost-over-Progress [1], GFG [2], EtE [3]) are competitive alternatives to the classical routing protocols for wireless *ad hoc* networks (AODV [4], OLSR [5]). Indeed, classical routing protocols exchange *O*(*n*^2^) messages for route discovery and require *O*(*n*) routing states at each node where *n* is the total number of nodes. On the other hand, in geographic routing protocols, nodes only need to store their and their neighbor’s coordinates.

Nevertheless, position information provided by devices is not always a feasible solution for sensor networks since GPS do not work in every environment. GPS are bulky, energy-costly and expensive. Without such positioning devices, the option is to assign nodes ’virtual’ geographical coordinates with an *internal location service*. These virtual coordinates do not necessarily embed global positioning information. They just have to be consistent to allow routing. Internal location services have already been studied in the literature. The first common approach proposed in VCap [6], JUMPS [7] or Gliders [8] consists in computing a distance based on node hop count from a set of landmarks to obtain a virtual position. This approach is easy to implement and performances are interesting in terms of stretch factor and energy efficiency for some of the algorithms cited above [9]. However, packet delivery is not guaranteed even if a route between the source and the destination exists. Indeed, several nodes may hold the same virtual coordinates and label uniqueness is required for guaranteeing delivery.

The authors of [10] propose an alternative approach. In LTP [10], labels are assigned to nodes by building a tree through a depth-first search on the network. Each node is assigned a label depending on its position in the tree. The routing paths are embedded in the labels. LTP guarantees the delivery but is not energy aware and may provide paths with a high stretch factor.

In this paper, we focus on designing an energy-aware and scalable routing protocol that guarantees delivery for sensor networks where nodes are not aware of any positioning information. We introduce HECTOR, a Hybrid Energy-effiCient Tree-based Optimized Routing protocol. HECTOR builds two sets of virtual coordinates: *(i)* virtual coordinates similar to the ones built in VCost, *i.e.*, based on a node hop count distances to landmarks and *(ii)* a set of labels as in LTP. The first set of virtual coordinates allows HECTOR to find a greedy path in the forwarding direction of the destination. The second set of labels prevents HECTOR from reaching a dead end and the routing from failing by maintaining low stretch factor paths. Based on these two sets of coordinates, a node holding a packet chooses its neighbor to forward the message in a Cost-over-Progress (COP [1]) fashion to save energy. The COP looks for nodes in the forwarding direction (here based on virtual coordinates or/and labels) and selects the one that minimizes the cost of transmission to this node over the progress made towards the destination.

HECTOR has the following properties:

- **Scalable:** Except the labeling steps which occurs at the bootstrap, to make a routing decision, a node has to be aware only of the location of itself, of its neighbors and of the final destination. Moreover, HECTOR is memoryless: no routing information has to be stored at the node and constant amount of information is embedded in the message along the path.
- **Loop free:** HECTOR is loop-free since it is a greedy routing that always makes any sender node *s* on the path forward to a node closer to the destination (in our coordinate system) than the sender node.
- **Guaranteed delivery:** HECTOR guarantees the delivery thanks to its set of labels derived from a tree. In the very worst case, HECTOR follows the tree that provides exactly one path between any pair of nodes.
- **Energy efficient:** HECTOR selects the node that minimizes the cost over the progress towards the destination. Simulations show its superiority over existing alternatives while guaranteeing delivery, and only up to 30% additional power compared to centralized shortest weighted path algorithm.

We then propose an extension of HECTOR based on multiple trees and theoretically prove the packet delivery. Simulations show that HECTOR provides fair performances regarding the energy efficiency and the path length. In addition, as far as we know, it is the first algorithm to propose a geographic routing protocol where nodes are not aware of their positions, which is both energy-efficient and guaranteed-delivery. Moreover, HECTOR does not rely on specific assumptions (e.g., Unit Disk Graph) or any radio propagation model. It may be applied in any general topology. For all these reasons, to our knowledge, HECTOR has no competing solutions. Indeed, classical routing protocols such as AODV [4] or DSR [11] trigger a flooding from each source while HECTOR provides a fixed amount of flooding (only at bootstrap) from all landmarks and tree root. Existing geographical protocols either need positioning system such as MFR [12] or GFG [2], do not guarantee delivery such as VCap [6] or VCost [9], or are not energy-aware like LTP [10].

The global analysis of HECTOR is performed by assuming that the network topology remains stable for at least the time needed to route a packet from its source to its final destination.

This paper is organized as follows. We briefly cover related work in Section 2. In Section 3, we present the way of assigning the two sets of coordinates and introduce our model and assumptions. We describe HECTOR in Section 4. The HECTOR extension to multiple trees is motivated in Section 5. Then, we compare HECTOR’s performances to existing methods in Section 6 by simulations and conclude in Section 7.

## 2. Related Work and Motivations

Routing in wireless sensor networks is a challenging task. Many different approaches have been proposed in the literature. We can identify three main classes of routing protocols: *(i)* proactive routing such as OLSR [5]*(ii)* reactive routing such as AODV [4] and *(iii)* geographic routing, or georouting. This latter approach is receiving more and more attention since it is a memory-less and scalable approach, unlike the two other ones. In a geographic approach, every node is aware of the exact or virtual coordinates (position) of itself, its neighbors and of the destination. Exact location coordinates may be available from GPS [13] or any other position mean [7].

Each of two families of georouting protocols (with exact and virtual coordinates) can be divided based on its properties with respect to the metric used (hop count or power), and whether or not it guarantees delivery. Therefore there are four classes of algorithms: *(i)* simple hop count based algorithms without guaranteed delivery, *(ii)* hop count based with guaranteed delivery, *(iii)* energy-efficient without guaranteed delivery or *(iv)* guaranteed-delivery and energy-efficient. Table 1 sums up the different categories and algorithms.

There are two well-known algorithms for the case where nodes are aware of their exact geographical coordinates available from GPS [13] or Galileo [15] or any estimation of them [16]. In *Most Forward Routing with progress (MFR)*[12], the node *S* currently holding the packet for destination *D* forwards it to neighbor *A* whose projection on line *SD* is closest to *D*. In *greedy* routing [14], *S* forwards the message to the node that is closest to *D*. These are simple localized algorithms that do not guarantee delivery. A packet can be trapped in a local minimum and the algorithms fail to find a path to the destination leading to low delivery rates. In dense networks the algorithms perform well.

Greedy georouting has then been enhanced in two directions, toward changing hop count to another metric, and toward providing guaranteed delivery. Power aware greedy routing algorithms were first studied in [17]. Instead of counting hops, power consumption on edges on a route was considered as the cost. An algorithm with general cost metric was proposed in [1]. Cost over Progress based routing [1] (COP) is a localized metric aware greedy routing scheme. A node forwards a packet to the neighbor closer to destination *D* such that the ratio of the energy consumed to the progress made (measured as the reduction in distance to *D*) is minimized. Though cost efficient, this algorithm does not guarantee delivery. Cost could be an arbitrary metric, such as hop count, power consumption, reluctance to forward packet, delay *etc*.

In [2], greedy routing is applied till reaching either the destination or a dead end. In latter case, face routing is applied to recover from failure. Face routing requires the network topology to be a planar graph (*i.e.*, no edges intersect each other). The graph planarization (through a Gabriel Graph [18] or a Relative Neighborhood Graph [19]) divides the graph in faces. The face that contains the line (*SD*), where *S* is the failure node, and *D* is the destination node, is traversed by right/left-hand rule (placing a virtual hand on the wall of the face) until a node *A* closer to destination than *S* is encountered. It has been shown in [2] that face routing guarantees recovery traversing the first face. Greedy routing continues from *A* until delivery or another failure node is encountered. GFG guarantees delivery but uses hop count as metric, and is therefore not energy-aware. Many georouting protocols guaranteeing delivery are only variants of GFG [3,20]. There also exist some beaconless georouting protocols based on the same idea [21].

We now describe approaches that rely exclusively on virtual coordinates, derived from either relative distances or hop counting to a set of landmark nodes in the network, without the intervention of external location services. The general idea is to define a virtual coordinate system and use it to induce a routing protocol based on the virtual coordinates. We survey some of them below (Jumps [7] or VCost [9]). A system of virtual coordinates based on three *landmarks* is proposed. Nodes are assigned a tuple of coordinates given as the number of hops the node is distant from each *landmark*. This virtual coordinate system establishment is described in detail in Section 3.1.

In VCap and JUMPS [7], nodes apply a greedy routing [14], based on the Hamming distance computed on these coordinates (instead of the Euclidean distance). The storage overhead for each sensor is limited to the storage of its coordinates and the coordinates of its neighbors. The authors show how the coordinate system is consistent for a given density of the network, *i.e.*, nodes with the same coordinates lie within a limited number of hops from each other. A different approach is used in [8] where landmarks are selected more carefully after partitioning the nodes into tiles, and elaborate gradient descent procedures are used to route packets, and high communication and storage overhead is required to increase the delivery rate. However these approaches are neither energy-efficient nor guaranteed-delivery. Therefore VCost [9] proposes to use this system by applying a greedy cost-over-progress routing, as in COP [1], still based on the Hamming distance. VCost is energy aware but still does not guarantee delivery.

Liu and Abu-Ghazaleh [22] observed that increasing the number of landmarks cannot eliminate virtual anomalies since some portions of the network may be 1-connected to the rest of network. They propose a one-dimensional virtual coordinate system based on depth first search (DFS) preorder traversal of the graph. Starting from a root node, nodes are labeled 1, 2, 3... with label assigned when a node is visited for the first time. Each node *m* also has an interval [m, q] starting from itself until all its children nodes are assigned, before traversal returns back to its parent. Routing is based on these labels. Current node may have few forwarding options; each of them is a neighbor that contains destination label within its interval of labels. Forwarding to a child node is favored to forwarding to parent node.

In LTP [10], the authors introduce a new coordinate system, based on a tree construction. Each node is assigned a label which embeds the path between this node to any other node in the network, based on the path in the tree which is unique. The labeling process of LTP is described in more details in Section 3.2. Because of this labeling, LTP ensures the delivery of the message and the success of the routing but is not energy aware and may provide paths which are much longer than the optimal ones.

In this paper, we propose a routing protocol that combines early results from the literature in order to provide a protocol routing that at the same time *(i)* is energy efficient, *(ii)* guarantees packet delivery and *(iii)* does not need any external position information but a means to estimate relative distance between neighboring nodes.

## 3. Preliminaries

Our routing process uses two sets of coordinates (*V*, *T*). *V*(*u*) is the set of coordinates of node *u* used to provide a progress in the geographic graph, limiting the stretch factor of the path length, but which cannot ensure the delivery if used alone. We use *V* coordinates based on landmark hop distances, as in VCost [9]. *T*(*u*) is the set of labels that allows guaranteed packet delivery, *i.e.*, if the network is connected, *T* coordinates provide a path between any pair of nodes. We use *T* coordinates as in LTP [10]. Each of these coordinates is associated to a distance: *d_V_* and *d_T_* respectively in order to measure a progress over each kind of coordinates. In the rest of this paper we will refer as “virtual coordinates” for *V* coordinates and to “labels” for *T* coordinates.

### 3.1. Building V Coordinates

**Figure 1.**
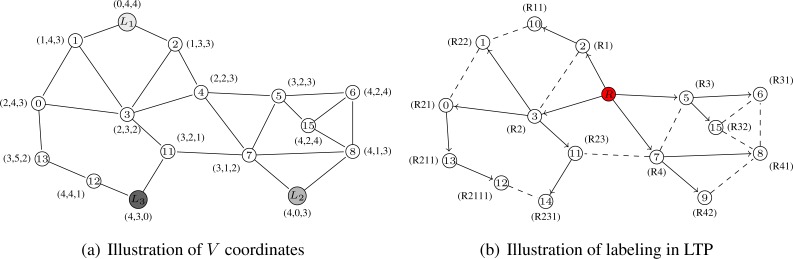
On Figure 1(a), the tree root is node 4 and has the label *R*. Node 13 is labeled *R*211 since it is the first child of node 0, which has label *R*21. Dashed lines represent physical links. On Figure 1(b), node 4 has coordinates (2, 2, 3) since it is 2-hop away from landmarks 1 and 2 and 3-hop away from landmark 3.

These coordinates are similar to the ones in VCap [6], JUMPS [7] or VCost [9]. Several nodes, *L*_1_, . . . , *L_k_* with *k* ≥ 3, in the network are distinguished as *landmarks*. Each landmark broadcasts a beacon in the network incremented at each hop. From it, an arbitrary node *x* knows its virtual coordinate vector *V*(*x*) = (*h*_1_, . . . , *h_k_*) where *h_i_* is the hop-distance between *x* and *L_i_*. Figure 1(a) shows an example of how nodes are assigned virtual coordinates. We suppose 3 landmarks: nodes 10, 9 and 14. Every node thus has a 3-dimensional vector as coordinates constituted by the number of hops between itself and every landmark. For instance, node 0 can reach Landmark 1 (node 10) in 2 hops, Landmarks 2 (node 9) in 4 hops and Landmark 3 (node 14) in 3 hops. Its virtual coordinate is thus *V*(0) = (2, 4, 3). The distance used on these virtual coordinates is *d_V_* where *d_V_* (*u*, *u*′) is the Hamming distance from node *u* to node *u*′ on *V* coordinates $\left(d_{V}\left(u,u\prime \right)=\sum_{i=1}^{M}\left|h_{i}\left(u\right)-h_{i}\left(u\prime \right)\right|\right)$. For example, on Figure 1(a), the distance *d_V_* (0, 8) between node 0 (*V* (0) = (2, 4, 3)) and node 8 (*V* (8) = (4, 1, 3)) is *d_V_* (0, 8) = |4 − 2| + |1 − 4| + |3 − 3| = 2 + 3 + 0 = 5.

Obviously, using only these coordinates does not guarantee delivery since the node coordinates are not unique (*i.e.*, several nodes may have the same virtual coordinates) and thus do not identify a single node. This is for example the case for nodes 6 and 15 on Figure 1(a) which are both labeled with (4, 2, 4).

### 3.2. Building T Labels

We build *T* labels in the same fashion as in LTP [10]. This labeling is performed through a tree construction. The tree is built iteratively from the root to the leaves. At bootstrap, a node is designed as root. This node may be a special node such as a fixed landmark. At each step, every freshly labeled node queries its unlabeled neighbors and then gives a label to each answering node. If *l*(*u*) is the label of node *u*, the *k^th^* neighbor of node *u* is labeled *l*(*u*)*k*. Figure 1(b) gives an example of how the nodes are labeled. The tree root is node 4 and has the label *R*. Node 13 is labeled *R*211 since is it the first child of node 0 which has label *R*21. The tree gives the shortest path in number of hops from the root to any other node. The distance used in the tree is based on label size and common prefix which can give the hop distance between any two nodes of the network. Thus the distance between node *a* and node *b* is *d_T_* (*a*, *b*) = |*l*(*a*) − *l*(*c*)| + |*l*(*c*) − *l*(*b*)| where *c* is the lowest common ancestor of *a* and *b* and *l*(*a*) is the label size of node *a*. From Figure 1(b) the distance between node 9 and node 5 is thus *d_T_* (9, 5) = |*l*(9) − *l*(4)| + |*l*(4) − *l*(5)| = |3 − 1| + |1 − 2| = 3.

As described in [10], the path is encoded in the labels. There exists a path encoded in node labels between any two nodes of the network. This path is the path in the tree, which, by definition, always exists and is unique (for *t* = 1).

### 3.3. Assumptions and Notations

Let *N*(*u*) be the set of physical neighbors of node *u*, *i.e.*, the set of nodes in communication range of node *u*. Let *δ*(*u*) be the cardinality of this set, also called the degree of node *u: δ*(*u*) = |*N*(*u*)|. We define *N_V_* (*u*, *u*′) as the set of neighbors of node *u* that reduce the distance to node *u*′, regarding the *V* coordinates: *N_V_* (*u*, *u*′) = {*v*|*v* ∈ *N*(*u*), and *d_V_* (*v*, *u*′) < *d_V_* (*u*, *u*′)}. Similarly, *N_T_* (*u*, *u*′) is the set of neighbors of node *u* that reduce the distance to node *u*′, in *T* coordinates: *N_T_* (*u*, *u*′) = {*v*|*v* ∈ *N*(*u*), *d_T_* (*v*, *u*′) < *d_T_* (*u*, *u*′)}.

Although HECTOR is cost model-independent, for the sake of proof of concept, we use the most common energy model [23], which is as follows: *cost*(*r*) = *r^α^* + *c* if *r* ≠ 0, 0 otherwise, where *r* is the distance separating two neighboring nodes, *c* is the overhead due to signal processing, and *α* is a real constant (>1) that represents the signal attenuation. Note that, in reality, this needs to be multiplied with a constant that includes, for example, the message length.

The optimal transmission radius, r*, that minimizes the total power consumption for a routing task is equal to: ${\text{r}}^{*}=\sqrt[{\alpha }]{\frac{c}{\alpha -1}}$ assuming that nodes can be placed on a line toward the destination [17].

Let us introduce the functions *COP_T_* and *COP_V_* as functions defining selection criteria of *s*’s next hop toward *d* in a cost-over-progress fashion [1] over coordinates *T* and *V* respectively. *s* selects node *b*, which minimizes *COP_T_* or *COP_V_* as defined later in Algorithm 1. These functions are as follows:

(1) $${\mathrm{COP}}_{T}\left(u,v,d\right)=\frac{\mathrm{cost}(|\mathrm{uv}|)}{d_{T}(u,d)-d_{T}(v,d)}$$

(2) $${\mathrm{COP}}_{V}\left(u,v,d\right)=\frac{\mathrm{cost}\left(\left|\mathrm{uv}\right|\right)}{d_{V}\left(u,d\right)-d_{V}\left(v,d\right)}$$

where |*uv*| is the Euclidean distance between nodes *u* and *v*.

Algorithm 1 formally describes this routing process.

**Algorithm 1:** Run at each node *u* on the routing path toward *d* to select next hop.

1:	**if** *u* = *d* **then**
2:	exit {/*Routing has succeeded*/}
3:	**else**
4:	*H* = {{*N_T_* (*u*, *d*)} ∪ {*v*|*d_T_* (*v*, *d*) = *d_T_* (*u*, *d*)}} ∩ {*N_V_* (*u*, *d*)}
5:	**if** (*H* = ∅) **then**
6:	{/*No node is closer to *d* than *u* on both *V* and *T*.*/}
7:	*H*′ = {*v*|*COP_T_* (*u*, *v*, *d*) = min_*w*∈*N*_*T*__ (*u*) *COP_T_* (*u*, *w*, *d*)}
8:	**else**
9:	*H*′ = {*v*|*COP_V_* (*u*, *v*, *d*) = min*_w_*_∈_*_H_COP_V_* (*u*, *w*, *d*)}
10:	**end if**
11:	**if** (|*H*′| > 1) **then**
12:	*Next_Hop* = *rand*(*H*′)
13:	**else**
14:	*Next_Hop* = *v* where *H*′ = {*v*}
15:	**end if**
16:	**end if**

In this paper, we assume every node is able to control its transmitting power (and thus its range) and to estimate the Euclidean distance between itself and every of its neighbor, based on the received signal strength (RSSI).

HECTOR uses RSSI rather than the angle of arrivals or triangulation that require additional communication overhead. In addition, even if some obstacles or external environment impact could mislead the computing of the distance based on RSSI, this computed distance reflects the state of the link. If a short link is seen as long by the node because of low RSSI, the link will be less likely to be used, which is a positive point. Virtual distances are not suitable in the cost calculation since they do not reflect the real cost of the transmission.

## 4. HECTOR

### 4.1. Algorithm Description

Each node *u* has two sets of coordinates (*V*, *T*) as defined in Section 3.

The routing algorithm combines advantages of both kinds of coordinates : *(i)* virtual coordinates as in VCost [9] allow the reduction of the path length and *(ii)* labels as in LTP [10] avoiding reaching a dead end and to guarantee delivery.

The basic idea is the following. A source node *s* holding a packet for a destination node *d* performs an energy-efficient greedy routing scheme in a VCost fashion. In order to avoid to be trapped in a local minima, the routing algorithm selects the next hop with regard to not only the virtual coordinates but also the labels. The routing process runs as follows. When node *u* receives a message for node *d*, it first considers its neighbors in the forward direction, based on both their labels and virtual coordinates. It only considers nodes *v* for which *d_T_* distance toward *d* is equal or smaller than the tree distance between *u* and *d* (*d_T_* (*v*, *d*) ≤ *d_T_* (*u*, *d*)). Such neighbors always exist (whenever source and destination nodes are connected) because of convergence of label-based routing. The algorithm first checks whether any one of these nodes also provides a progress with respect to landmark coordinates. Let *H* = *N_T_* (*u*) ∩ {*N_V_* (*u*) ∪ *v* |*d_T_* (*v*, *d*) = *d_T_* (*u*, *d*)} be the set of such nodes.

If *H* ≠ ∅, then *u* selects its next hop among the nodes in *H* (thus reducing the distance toward the destination regarding coordinates *V* and not increasing distance regarding *T* labels) as the node *v* that provides the best ratio cost over progress to the destination regarding the virtual coordinates (*v* such that *COP_V_* (*u*, *v*, *d*) = min_*w*∈*N_V_*_ (*u*) *COP_V_* (*w*)).

Otherwise (that is, if *H* = ∅), the node selects its neighbor *v* that provides the best ratio cost over progress (as in [1]) to the destination regarding the labels (*v* such that *COP_T_* (*u*, *v*, *d*) = min_*w*∈*N_T_*_ (*u*) *COP_T_* (*u*, *w*, *d*)). Such a node always exists since there always exists exactly one path in the tree between any two nodes. In case of ties, the next hop is chosen at random between candidates.

### 4.2. Algorithm Quality

**Lemma 1** *A packet cannot transit from a node u to another node v if V* (*u*) = *V* (*v*) *(or if d_V_* (*u*, *d*) *< d_V_* (*v*, *d*)*) unless there is an absolute progress regarding T labels (if d_T_* (*u*, *d*) > *d_T_* (*d*, *v*)*)*.

Proof Let us assume that node *u* holds a packet for a destination *d*. Suppose that nodes *u* and *v* have the same *V* coordinates (*V* (*u*) = *V* (*v*)) or that *v* is farther than node *u* regarding *V* coordinates (*d_V_* (*u*, *d*) < *d_V_* (*v*, *d*)). Then *v* ∉ *N_V_* (*u*, *d*), which means that *v* ∉ *H*. The selected next hop thus belongs to *H*′ = {*v*|*COP_T_* (*u*, *v*, *d*) = min_*i*∈*N_T_*_ (*u*) *COP_T_* (*u*, *i*, *d*)}, which contains every neighbor of *u* closer to *d* than *u* regarding *T* labels. Thus, if node *v* is chosen as the next hop, that means that *v* ∈ *H*′ and thus provides a progress regarding *T* labels. Note that in the worst case (*i.e.*, when the progress on *T* labels is minimal), the next hop is either the parent or a child of node *u*.

**Lemma 2** *The routing protocol described in Algorithm 1 is loop free.*

Proof We introduce an order among all nodes with respect to combined distance to destination *d*. Consider *d_T_* (*u*, *d*) as the primary key, and *d_V_* (*u*, *d*) as the secondary one. Two nodes are sorted by their primary key. In case of ties, the secondary key is used. Thus *u* < *v* if and only if *d_T_* (*u*, *d*) ≤ *d_T_* (*v*, *d*) and (*d_V_* (*u*, *d*) < *d_V_* (*v*, *d*) or *H* = ∅). Let us assume that node *u*_0_ is the source of a packet, *d* its destination and node *u*_1_ the next hop chosen by node *u*_0_. If *H* ≠ ∅ then *d_T_* (*u*_1_, *d*) ≤ *d_T_* (*u*_0_, *d*) as per restriction. Also similarly *d_V_* (*u*_1_, *d*) < *d_V_* (*u*_0_, *d*). Therefore *u*_1_ < *u*_0_ in our order. Let *H* = ∅. Then *d_T_* (*u*_1_, *d*) < *d_T_* (*u*_0_, *d*) and therefore again *u*_1_ < *u*_0_. Our routing process therefore strictly reduces distances to destination regarding *T* coordinates at every step in the order defined by given primary and secondary keys. This means that loops cannot be created.

**Lemma 3** *In the routing protocol described in Algorithm 1, there always exists a next hop that is closer to the destination regarding both sets of virtual coordinates.*

Proof Let us consider a source *u* and a destination *d*. By construction, if a node in *N_V_* (*u*, *d*) is chosen as the next hop, this ensures a progress on the *V* coordinates. If the next hop is chosen in *N_T_* (*u*, *d*) this ensures a progress in the tree toward the destination. The progress will occur since *N_T_* (*u*, *d*) is a nonempty set.

It is worth noting that the progress made on *V* is more important than the progress made on *T* labels in the geographical space. Indeed, the next hop in the *T* labels can have the same *V* coordinates and thus more or less the same Euclidean distance to the destination. These lemmas show that the routing protocol HECTOR described in Algorithm 1 always works in a greedy way. The greedy aspect provided by this algorithm makes it simple, memoryless and scalable.

**Theorem 4** *The routing algorithm described in Algorithm 1 guarantees delivery.*

Proof Each node has a unique label due to the labeling process described in Section 3. This ensures that the destination of a packet is unique and that at each step of the routing protocol, a next hop closer to the destination can be found. Based on Lemmas 1, 2 and 3, if a path exists (if the network is connected), the routing protocol will find it in a greedy way.

## 5. Multiple Trees Hector Extension

As we could see, in Hector, the packet delivery is guaranteed because of the use of a tree. Nevertheless, following that tree may lead to important stretch factors in the routing path. One way to bypass this drawback is to use multiple trees. All trees are built independently as explained in Section 3.2. Each node has one label per tree. The *T* label of a node *u* is now: *T* (*u*) = {*l_i_*(*u*)}_*i*=0,..,*t*−1_ where *t* is the number of trees and *l_i_*(*u*) is the label of node *u* in Tree *i*.

**Figure 2.**
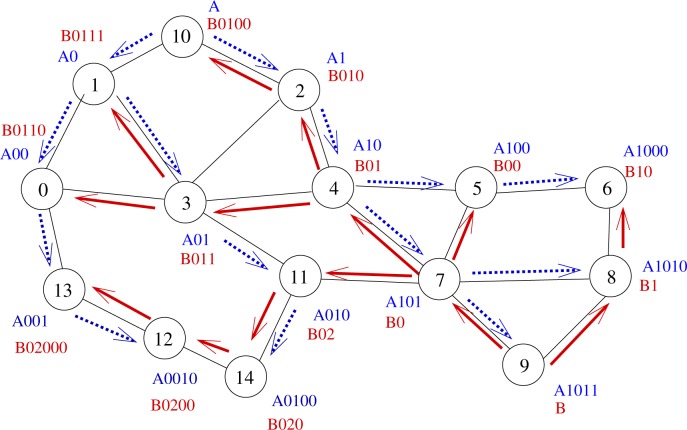
Illustration of the use of multiple trees.

A 2-tree example is displayed by Figure 2. Let us illustrate on this example what advantages may bring the use of several trees. Let us assume that node 0 wants to send a message to node 6. The shortest path in the graph from node 0 to node 6 is 0 − 3 − 4 − 5 − 6 (path length: 4 hops). If we use Tree *A* (blue tree), the message will follow labels *A*00 − *A*0 − *A* − *A*1 − *A*10 − *A*100 − *A*1000, which corresponds to a 6-hop path going through nodes 0 − 1 − 10 − 2 − 4 − 5 − 6. If we use Tree *B* (blue tree), the message will follow labels *B*0110 − *B*011 − *B*01 − *B*0 − *B*1 − *B*10, which corresponds to a 5-hop path going through nodes 0 − 3 − 4 − 7 − 8 − 6. Note that using Tree *B* allows the use of a shortcut between nodes 7 and 8.

Hence, the use of several trees allows the use of more routes, which provides a better load balancing and shorter paths. In our example, node 0 will follow Tree *B* since it provides a shorter path than Tree *A*.

The use of several trees may even allow even shorter paths since the choice of the tree is performed independently at each routing step. If we look back at our example, node 0 computes the distance between each neighbor of its and the destination on every tree. It finds out that it has to send the message through Tree *B* to node 3. Node 3 runs the same algorithm and sends the message to node 4, still through Tree *B*. When node 4 has to elect the next hop to the destination, it finds out that the path is shorter by following Tree *A* and sends the message to node 5, which delivers the message to node 6. By switching dynamically and naturally between trees all along the path from the source to the destination, a 4 − *hop* path is followed (through nodes 0 − 3 − 4 − 5 − 6).

This is the motivation of multiple-tree HECTOR.

Note that building several trees bring obviously better performances but also presents a higher costs linked to the construction and maintenance of several trees. The evaluation performed in Section 6.4 shows the trade-off to adopt between cost and performance.

### 5.1. Algorithm

For using multiple trees, some additional notations are introduced.

Let Ω be the set of trees. We note *d_mT_* (*u*, *v*) the distance in the forest or set of trees between nodes *u* and *v*:

$$d_{\mathrm{mT}}\left(u,v\right)=\min_{t\in \Omega }d_{T}\left(u,v,t\right)$$

where *d_T_i__* (*u*, *v*) if the *d_T_* distance in tree *i* between nodes *u* and *v*. We note *N_mT_* (*u*, *u*′) the set of neighbors of node *u* that provides a positive progress to *u*′ in the forest: *N_mT_* (*u*, *u*′) = {*v*|*v* ∈ *N*(*u*), *d_mT_* (*v*, *u*′) < *d_mT_* (*u*, *u*′)}.

Based on this, we can now define the *COP_mT_* function as the cost over the progress realized over the forest and not a single tree as follows: ${\mathrm{COP}}_{T}\left(u,v,d\right)=\frac{\mathrm{cost}\left(\left|uv\right|\right)}{d_{\mathrm{mT}}\left(u,d\right)-d_{\mathrm{mT}}\left(v,d\right)}$.

By replacing 1-tree notations by these new notation, the same algorithm as Algorithm 1 applies. Algorithm 2 details the new routing algorithm. The current node *u* holding the packet first considers the set of its neighbors that provide both a positive progress on *V* coordinates and a positive or null progress over *T* coordinate whatever the tree considered (*u* considers nodes *v* such that *v* ∈ {{*N_mT_* (*u*, *d*)} ∪ {*v*|*d_mT_* (*v*, *d*) = *d_mT_* (*u*, *d*)}} ∩ {*N_V_* (*u*, *d*)}) and chooses the one among them that provides the best cost-over-progress over *V* coordinates. If no such node exists, *u* chooses the next hop as the one that provides the best progress over *T* coordinates over every tree. To break ties, it applies the *minimizing label* and *label balancing* rules that we describe below.

**Algorithm 2:** Run at each node *u* on the routing path toward *d* to select next hop.

1:	**if** *u* = *d* **then**
2:	exit {/*Routing has succeeded*/}
3:	**else**
4:	*H* = {{*N_mT_* (*u*, *d*)} ∪ {*v*|*d_mT_* (*v*, *d*) = *d_mT_* (*u*, *d*)}} ∩ {*N_V_* (*u*, *d*)}
5:	**if** (*H* = ∅) **then**
6:	{/*No node is closer to *d* than *u* on both *V* and *T* for all trees.*/}
7:	*H*′ = {*v*|*COP_mT_* (*u*, *v*, *d*) = min_*w*∈*N*_*mT*_ (*u*)_ *COP_mT_* (*u*, *w*, *d*)}
8:	**else**
9:	*H*′ = {*v*|*COP_V_* (*u*, *v*, *d*) = min*_w_*_∈_*_H_COP_V_* (*u*, *w*, *d*)}
10:	**end if**
11:	*M* = {*v*|*v* ∈ *H*′ ∩ |*l*(*v*, *t*)| = *min_t_*_′∈Ω,_*_w_*_∈_*_H_*_′_ |*l_t_*_′_ (*w*)|} {Minimizing label rule}
12:	*M*′ = {*v* such that *v* has the best balanced labels over nodes in *M*} {Label balancing rule}
13:	
14:	*Next_Hop* = *rand*(*M*′)
15:	**end if**

Indeed, some cases may appear in which, from the local point of view of the current node, every tree provides the same progress to the destination. For instance, let us consider node 13 on Figure 2 aiming to send a message to node 6. Here, paths in both trees *A* and *B* have the same length, regarding *T* coordinates (7 hops). Nevertheless, the message may follow the path through nodes 13−0−3−4−5−6, which is the shortest one but only if node 13 chooses Tree *A*. To help node 13 make the right decision, two selection rules are introduced: the *minimizing labels* rule and the *label balancing* rule.

*Minimizing labels* rule: When paths are equivalent, the next hop in route selection is the node with the lowest label size. The idea here is that by selecting the next hop in such a way, the message goes at least 1 hop toward the tree roots and, as further stated by Theorem 6, the path in the tree from the root to any other node is the shortest path. This is easily done by counting and summing the number of digits of every label of a node. For instance, node 13 has to choose between node 0 (which label global size is |*A*00| + |*B*0110| = 3 + 5 = 8) and node 12 (which global label size is |*A*0010| + |*B*0200| = 5 + 5 = 10). Thus here, Tree *A* is selected since the number of digits of node 0 is the smallest one. When node 0 is reached, it reiterates the same process and so on.

*Minimizing label* rule: This rule allows the selection of a node with a high position in every tree. This is interesting when the routes from the source to the destination have to pass through a parent in both trees.

Nevertheless, these two rules do not prevent from having nodes choosing at random, as it is the case if node 0 handles a packet for node 6. It has the choice between Tree *A* through node 1 and Tree *B* through node 3 which both offer a path of length 6 hops and which both have a global label size equal to 7. So, to break that final tie, we apply the *Label balancing* rule. This rule will favor the node which label sizes are load balanced. Indeed if label sizes are load balanced, that means that the root of the trees are more likely to be bypassed, which may prevent from contention. In this case, node 0 will choose node 3.

### 5.2. Algorithm Quality

We now prove that Algorithm 2 finds the shortest path in the forest.

**Definition 1** (**shortcut**) *We call shortcut a link between two distinct branches of the tree on the routing path.*

**Definition 2** (*t*-**shortcut**) *A t-shortcut is a link on a routing path that allows the switching from a tree to another one.*

**Lemma 5** *We assume that the network is stable at least during a minimum time that ensures that a packet can be routed from its source to its final destination. If the MAC layer and the Physical layer are ideal (no packet loss or interferences), there is no t-shortcut between a parent and a child.*

Proof Let us assume 3 nodes with labels *A*, *A*1 and *A*11. Let us assume that a *t*-shortcut exists between nodes *A* and *A*11 from another tree. This means that node *A*11 did not receive the *Discovery* request from node *A*, which is impossible since we assume that there is no message loss.

Lemma 5 can easily be extended to *t*-shortcuts between nodes with labels of size *X* and labels of size *X* + 2 resp.

We now give two definitions for a subtree. Definitions 3 and 4 are equivalent.

**Definition 3** (**Subtree**) *Node u belongs to the subtree of node v iff l*(*u*) ⊂ *l*(*v*).

**Definition 4** (**Subtree**) *Node u belongs to the subtree of node v iff node v is on the path in the tree from node u to the tree root.*

**Theorem 6** *If the MAC layer and the Physical layer are ideal, which means that there is no message loss, a shortest path to a destination node in the subtree of the source is the path which follows the tree.*

Proof This is true based on Lemma 5, because there is no possible *t*-shortcut in the same subtree.

This theorem means that, if a path from a node to another node in the same subtree exists, this path (by using this tree) is the shortest path. The two previous theorems mean that the *t*-shortcut and the *minimizing labels* rules are interesting when the destination is not in the subtree of the source. The *minimizing labels* rule is thus important when the destination is not in the subtree of the source (for all trees) because when the labels are minimized, it means that in at least 1 tree the message is forwarded toward the root of this tree.

## 6. Simulation Results

This section presents the simulation results of our algorithm. We compare our solution to the geographical algorithms of the literature that assume no position information: VCost [9], which is the best algorithm known regarding energy-efficiency, and LTP [10], which is one of the first known to guarantee delivery. In order to further evaluate the energy saving contributions of HECTOR, we also compare it to its variant HECTOR’, which selects as the next hop the node that maximizes the progress towards the destination (*i.e.*, it considers that *cost*(|*uv*|) = 1 ∀ *u*, *v* and tries to minimize *COP_T_* or *COP_V_*). HECTOR’ guarantees delivery but uses hop count as metric and is not energy aware. We first present the simulation setup and then give some performance results about energy consumption overhead, mean path length and mean hop length.

### 6.1. Simulation Setup

As we focus our performance evaluation study on network layer mechanisms, for our performance results to be independent of the lower layers, we chose to use our home-made simulator that assumes ideal MAC (no packet collision, no delay) and Physical layers (no interference, BER = 0, isotropic radiation pattern). The network can be described as follows. Nodes are deployed in a 1,000 × 1,000 square following a two dimensional Poisson Point Process with different intensities *λ*. In such a Poisson Point Process, the total number of nodes is probabilistic and is obtained from a Poisson Law of intensity *λ*, which is correlated to the mean node degree $\delta :\lambda =\frac{\delta }{\pi R^{2}}$. Nodes are uniformly distributed over the area. Nodes can adapt their range between 0 and *R* = 200. We only consider connected networks.

**Figure 3.**
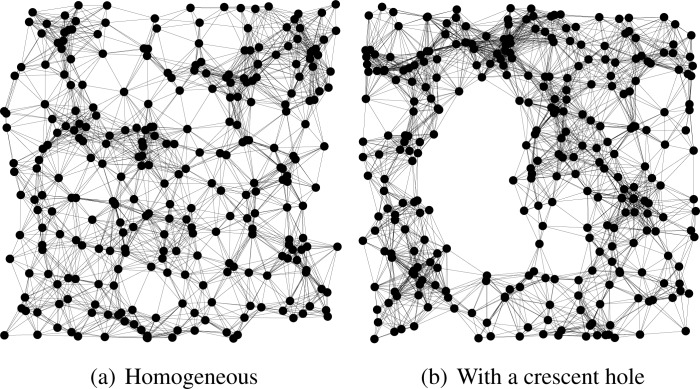
Network topologies.

We compare HECTOR, LTP [10] and VCost [9] for the same samples of node distribution, same source and destination pairs, both randomly chosen. Landmarks and the tree root are randomly chosen among the nodes. Finally, to show the impact of the use of the two sets of coordinates over the guaranteed delivery, we evaluate the performances of the routing schemes over a homogeneous network and over a topology with a crescent hole (see Figure 3).

All results are the average of statistics retrieved from more than 100 simulation runs and meet a 98% interval. Note that the bootstrap cost induced by the coordinates setting is not integrated in these results. We keep for future work the evaluation of this cost and the maintenance of the virtual coordinates.

We evaluate the energy consumption overhead (ECO) of each algorithm based on the energy model described in Section 3. As in [23], we use *c* = 10^7^ and *α* = 4, which leads to an optimal range of r^*^ = 100 [24]. To further evaluate the routing protocols, we computed their energy overhead using as reference the optimal centralized energy weighted shortest path (SP) (Dijkstra algorithm [25]). We let *e_i_* and e^*^ be the energy consumed using any described protocol and the centralized SP protocol, respectively. We define the energy overhead as the ratio $\frac{e_{i}-e^{*}}{e^{*}}\times 100$. Note that 0% overhead means that the algorithm consumes the same energy than the optimal algorithm.

We also evaluate the mean path length and mean hop length obtained for each protocol and give visual results of routing process.

### 6.2. HECTOR Results

**Figure 4.**
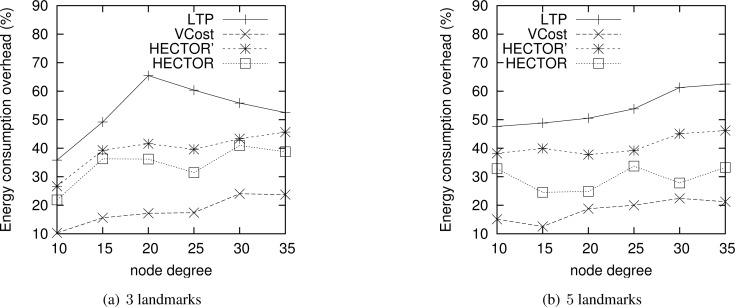
ECO when VCost succeeds for 3 and 5 landmarks for a homogeneous topology.

**Figure 5.**
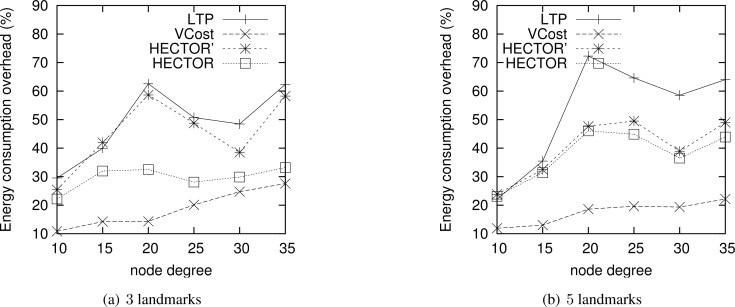
ECO when VCost succeeds for 3 and 5 landmarks for a topology with a hole.

**Energy consumption overhead when VCost succeeds.** Figure 4 shows the ECO for paths provided by the different algorithms when VCost succeeds for a given source-destination pair. The energy overhead is drawn depending on the mean node degree and on the number of landmarks used to build *V* coordinates. We can see from this figure that HECTOR provides the lowest overhead within the protocols that guarantee delivery. We can see that the node degree and the number of landmarks have a limited impact on the performances of each protocol since the figure shows the energy overhead once a path has been found. Since the environment is homogeneous, the impact of these parameters is thus negligible on the path features. Figure 5 shows the same results in a topology with a hole, still when VCost succeeds. Here we can also see that the performances of HECTOR are the best within the protocols that guarantee delivery.

As expected, for each case, HECTOR provides a greater overhead than VCost. This is due to the routing process in HECTOR that tries to provide a progress in the tree at any step. Therefore, the tree root position is important for minimizing the energy consumption for a given source and destination, but it is not possible to have an optimal tree root position for all possible source-destination pairs.

**Figure 6.**
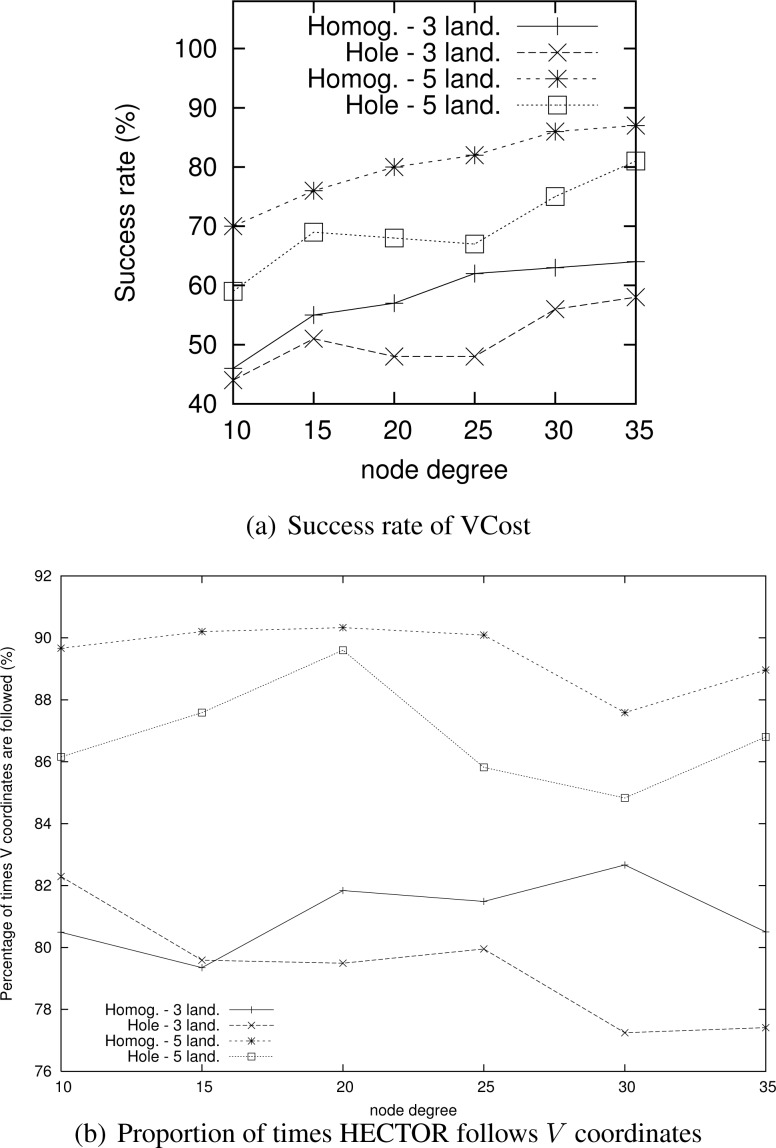
Success rate of VCost routing algorithm (**a**) and number of times HECTOR follows *V* coordinates (**b**) for each scenario.

Nevertheless, as Figure 6(a) shows, the success rate of VCost is far from 100% and is not the same following the different scenarios. We can note that the more VCost succeeds, the more HECTOR is energy-efficient and sticks to VCost performances. This is because, in Algorithm 1, the *V* coordinates are chosen uppermost. On the other hand, the less VCost succeeds, the more HECTOR sticks to LTP performance. If VCost fails, that means that there is no path following *V* coordinates and thus HECTOR algorithm follows *T* labels to ensure packet delivery, as in LTP. This is also confirmed by results displayed by Figure 6(b) which are the percentage of times HECTOR progresses over *V* coordinates rather than only on *T* labels. This is correlated with the success rate of VCost which only follows *V* coordinates.

Note that an exception occurs for low densities of HECTOR with 3 landmarks. This is due to, by construction and because of the low densities, the path followed by VCost can be far from the label root, which forces HECTOR not to follow the VCost coordinates but the LTP labels. This is less the case in topologies with holes because VCost coordinates also bypass the hole, which make the path followed by VCost closer to the root. This explains this phenomenon. We integrated this explanation in the revised version.

**Figure 7.**
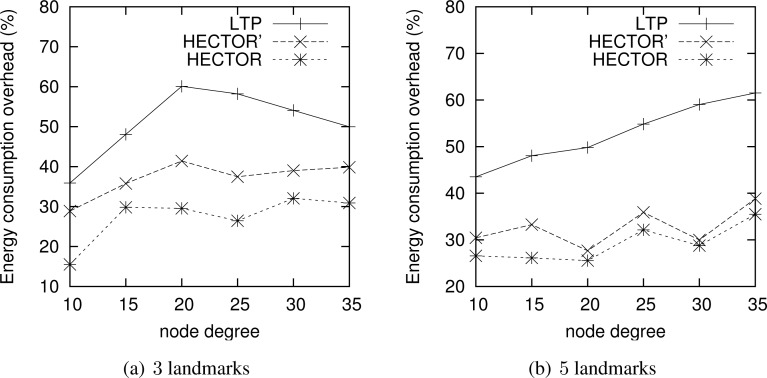
ECO when VCost fails for a homogeneous topology.

**Figure 8.**
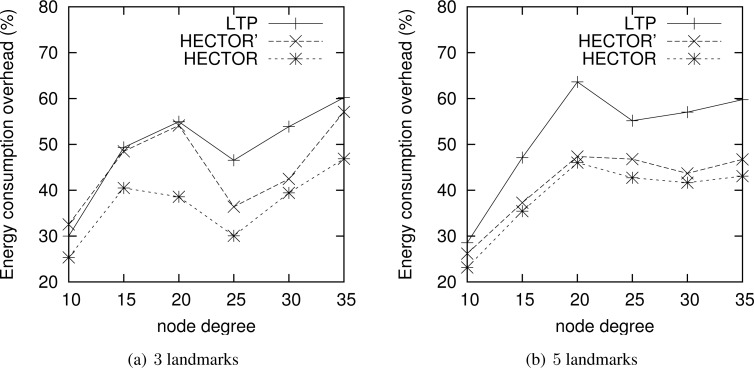
ECO when VCost fails for a topology with a hole.

**Energy consumption overhead when VCost fails.** When VCost fails, HECTOR has to follow *T* labels to reach the destination. This feature is one of the main contributions of HECTOR and cannot be observed in Figures 4 and 5 since the latter ones show results for simulation runs where VCost succeeds. Therefore, Figures 7 and 8 draw the results of HECTOR, HECTOR’ and LTP for every simulation run, about paths on which VCost fails.

In Figures 7 and 8, HECTOR is the algorithm that provides the best performances regarding energy consumption, followed by HECTOR’. LTP, once again, is the least performing algorithm. Moreover, as expected, we can note that the global behavior of HECTOR and HECTOR’ is the same as LTP’s. As already mentioned, this is because, when there is no progress over *V* coordinates, HECTOR and HECTOR’ follow the *T* labels, as LTP, and so on till reaching a node which can provide a progress regarding *V* coordinates.

**Figure 9.**
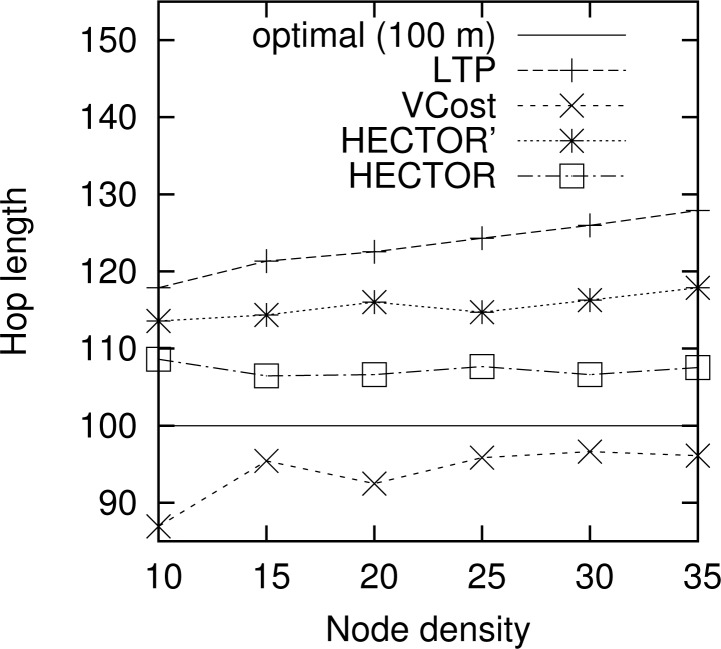
Mean hop length.

**Hop length.** Figure 9 shows the mean hop length along the routing path for every algorithm. The optimal hop length (based on energy consumption) is plotted as a reference. Results are similar for other choices of the number of landmarks and the topology. We can notice that VCost and HECTOR follow edges whose lengths are close to the optimal one [17] in every case. The mean hop length is greater than the optimal one with HECTOR because the choice of the next hop is conditioned by the progress made over *T* labels, which leads to greater hop length because of tree construction. Indeed, because of the labeling process, close nodes are mainly at the same level in the tree and thus the progress they provide is null. Therefore, since nodes try to minimize the cost over progress ratio, they generally try to maximize the progress, thus reaching farther nodes.

**Figure 10.**
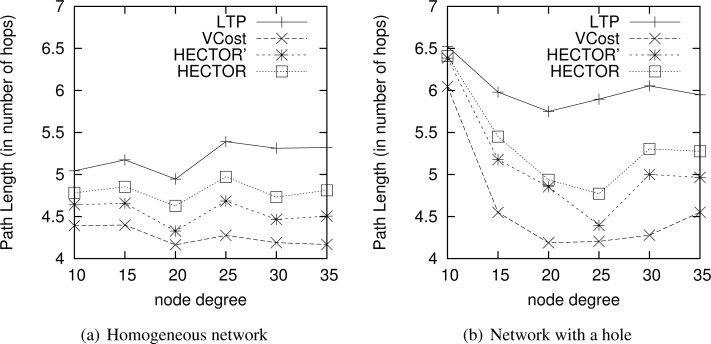
Path length in number of hops when VCost succeeds (3 landmarks).

**Path Length.** Figure 10 draws the path length in number of hops when VCost succeeds. We can notice that VCost is the algorithm that provides the shortest paths. This is because it is the only algorithm to select the next hop in the forwarding direction at each hop. LTP is the one achieving the longest paths since it follows the path in the tree, sometimes with shortcuts between branches but which is rarely the shortest path. The tree construction affects the mean hop length of LTP. The impact of having an energy efficient tree or a tree with optimized range is left to future works. As already mentioned, HECTOR tries to stick to VCost when it is possible. HECTOR’ acts as VCost but since it is not energy-aware, HECTOR’ takes long edges and thus gets shorter paths than HECTOR. Note that in the worst cases when HECTOR can never provide a progress over *V* coordinates and that there is only one candidate that provides a progress over *T* labels, it also follows the tree. In this latter case, the path length provided by HECTOR is longer than the one achieved by LTP since they both follow more or less the same route, but LTP takes longer edges and HECTOR tries to fit the optimal range.

**Figure 11.**
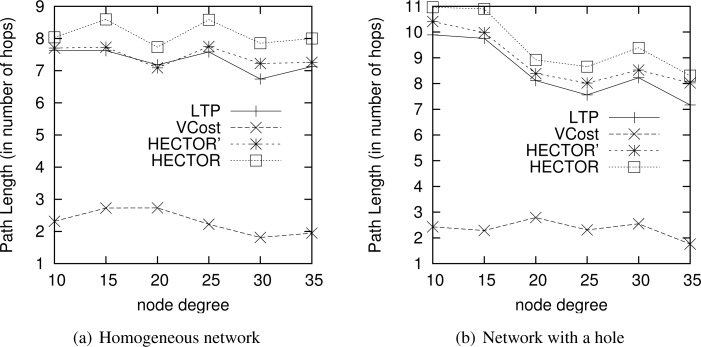
Path length in number of hops when VCost does not succeed. For VCost: number of hops before failing (3 landmarks).

When VCost fails, HECTOR and HECTOR’ make decision based on *T* labels only, and thus HECTOR and HECTOR’ stick to LTP. Thereby, HECTOR provides longer paths than LTP and HECTOR’, which are not energy aware and take long links while HECTOR favors edges less energy-costly. This behavior is highlighted in Figure 11, which shows the path length in number of hops when VCost fails.

Another interesting feature to point out is that globally, HECTOR provides longer paths than HECTOR’ and VCost while it spends less energy. This also shows that HECTOR distributes the energy spending over the nodes on the paths.

**Figure 12.**
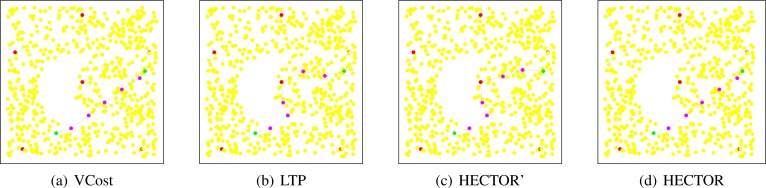
Illustration of the paths followed by each algorithm with the use of 5 landmarks. Source is in the right side. VCost and HECTOR follow the same path while LTP and HECTOR’ passes through the tree root.

**Path shapes.** Figure 12 shows example of the paths followed by each protocol in a network with a crescent hole. Five landmarks are randomly chosen and the tree root is the red/black node in the middle of the network. Source and destination are also randomly chosen. These schemes clearly show the behavior of each algorithm.

We can see in Figure 12 that VCost and HECTOR follow exactly the same path. This means that every hop provides a progress on both *V* and *T* coordinates. It is nevertheless worth noting that this would not appear in the general case. Indeed, for HECTOR to follow exactly the same path that VCost, a progress has to be made at each step on both sets of coordinates. Even if a progress is made on *V* coordinates by a node *u*, to be chosen, this node *u* has to also provide a progress on *T* labels, which is strongly related to the tree root, the source and the destination position. In the contrary, HECTOR’ does not try to minimize the COP and thus the first hop is different from that in VCost and directs it toward the hole. From it, in order to provide a progress regarding *T* labels and avoiding the dead end, HECTOR’ has to follow the path in the tree, as in LTP. The path followed by LTP goes trough the tree root which, in this particular case, increases the path length.

**Figure 13.**
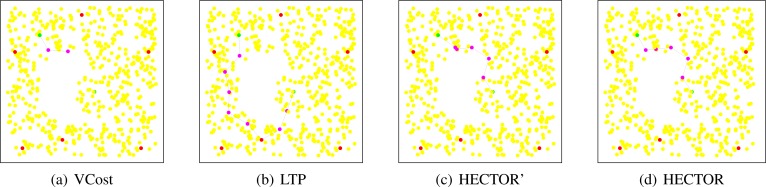
Illustration of the paths followed by each algorithm with the use of 5 landmarks. VCost fails after the second hop, LTP passes through the tree root and HECTOR combines both *T* and *V* coordinates.

In Figure 13, the same simulation is run with different source and destination pairs. The tree root is also modified. We can see from this figure that the path followed by VCost falls into a dead end after the second hop. One may think that when VCost fails, HECTOR follows the path of LTP. We can see in Figure 13(d) that HECTOR first follows the *V* coordinates and then avoids the dead end encountered by VCost by using both *T* and *V* coordinates. This example shows how the combination of both *T* and *V* coordinates can guarantee delivery and optimize the path length. Once again, LTP follows the complete path in the tree and provides a very long path.

It is worth noting that the landmarks and the tree root positions have a great impact on the routing process. In VCost, landmarks position may affect the success rate. In LTP, the tree root position may increase the path length, and in HECTOR, the path may be different depending on these positions.

### 6.3. Enlarging the Network

Till now, we have evaluated HECTOR by comparing it to other existing algorithms by running them in a restricted area and by making the node density grow. In this section, we fix the node density to *δ* = 15 and the maximum node range radius to *R_max_* = 200 and expand the network area size.

Indeed, in such a scenario, the energy consumption will necessary grow since nodes may be further one from the others and more hops are needed to connect them than in previous scenarios. This section allows us to check the scalability of HECTOR (in terms of growing area rather than increasing node density) in very large networks by being sure that we still ensure a low ECO.

**Figure 14.**
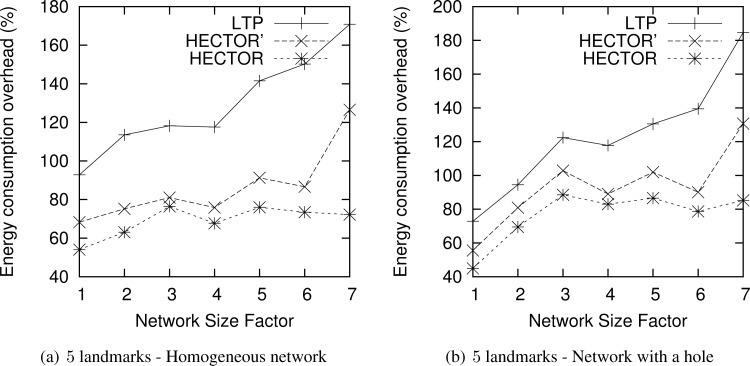
ECO when the network grows.

Figure 14 draws the ECO of the routes taken by each algorithm when the network size grows, for 5 landmarks. The results are similar for 3 landmarks. The abscissa axis plots the factor by which the network size has been multiplied. We only plot results where VCost fails since these runs represent the most energy costly results and the longest paths, as seen in previous sections.

One can notice that for homogeneous networks, even if the network grows as well as the route length, the energy overhead compared with the optimal shortest path consumed by HECTOR grows slowly with the network size. This is because HECTOR can follow *V* coordinates, and thus can have an energy consumption close to the optimal. Therefore, the energy consumed by paths followed by each algorithm is within a constant factor of the optimum. Also note that for distributions with a hole, the energy overhead tends to increase with the network size. This is due to the fact that the bigger the network (and longer the routes), the more likely greedy routing encounters a dead end and thus HECTOR has to follow the *T* labels and the tree, which gives longer paths and thus consumes more energy.

### 6.4. Multiple Trees

**Figure 15.**
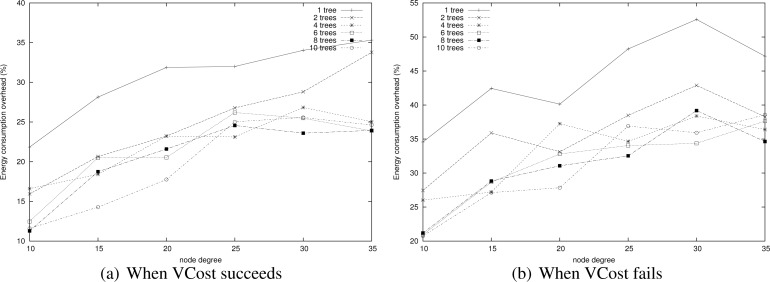
Energy consumption overhead.

We now measure the benefit of using multiple trees for HECTOR as described in Section 5. Indeed, paths is tress should be shortened but in return, this means that there are several trees to maintain and there is a cost. To do so, we compare the energy consumption of paths found by HECTOR with different numbers of trees, both when VCost succeeds and when it fails. Tree roots are spread randomly in the network. Results are displayed in Figure 15.

We can notice that globally, the energy consumption is lower when VCost succeeds. This is still because HECTOR is more likely to follow the VCost coordinates. What is interesting to notice is that in both cases, using two trees instead of one allows the great enhancement of the energy consumption while using more than two trees does not bring a lot, since energy consumed by HECTOR is globally the same whatever the number of trees. When the network is sparse, it can be worth using a third tree but this is only for some low density scenarios.

**Figure 16.**
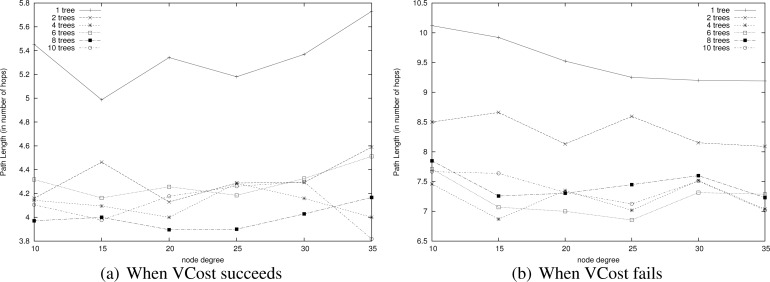
Path length.

This means that two trees are enough to find appropriate paths by switching from one tree to another one. This is confirmed by results shown in Figure 16 that displays the path length of each variant. Path length is similar for every variant that has two trees or more. These figures show that there is no need to build more than 4 trees since the energy saved is negligible. This is due to the fact that 4 trees are enough to find a direct way in the graph.

## 7. Conclusion

In this paper, we introduce HECTOR, a Hybrid Energy-effiCient Tree-based Optimized Routing protocol. HECTOR is a geometric routing protocol designed for wireless sensor networks. Unlike the approaches proposed in the literature, HECTOR is *(i)* based on virtual coordinates, *(ii)* energy aware, *(iii)* guarantees delivery, *(iv)* scalable and *(v)* do not assume any propagation radio model such as the Unit Disk Graph. These properties are provided by the combination of two sets of virtual coordinates used in HECTOR: landmark-based coordinates and tree-based coordinates. We proved the packet delivery and proposed some extension. Simulation results show that HECTOR exhibits fair performances compared to the protocols presented in the literature, regarding energy consumption and stretch factor. Analysis of the extension to multiple trees shows that with the use of only a single additional tree, performances could be enhanced even more. Moreover, as far as we know, HECTOR is the first geographic routing protocol based on virtual coordinates that is both energy-efficient and with guaranteed delivery. Note that in this paper we use landmark-based coordinates for the energy efficient step, but any other coordinate system may be used instead, including GPS localization. Therefore, we intend to explore another coordinate system other than the landmark-based one in order to avoid the preprocessing flooding step by applying dominating set [26].

The next step of this work is to provide a more reliable way to build the tree coordinates in HECTOR. Indeed, a weakness of HECTOR is due to the underlying tree(s) used for one set of coordinates. Building a tree with energy-aware properties would make HECTOR even more efficient. At last, other aspects to analyze are the study of HECTOR towards node mobility, asymmetric links and extension to heterogeneous networks [27].

## Figures and Tables

**Table 1.** Classification of Georouting Protocols.

	Exact Position	Virtual Position
hop count (HC)	MFR [12], greedy [14]	VCap
Energy-efficient (EE)	COP [1]	VCost [9]
HC+Guaranteed-delivery (GD)	GFG [2]	LTP [10]
EE+GD	EtE [3]	HECTOR
